# Flow cytometric analysis of T lymphocytes and cytokines in aqueous humor of patients with varicella zoster virus-mediated acute retinal necrosis

**DOI:** 10.1186/s12886-021-01951-1

**Published:** 2021-05-01

**Authors:** Hao Kang, Yunbo Wei, Ming Liu, Di Yu, Yong Tao

**Affiliations:** 1 Department of Ophthalmology, Beijing Chaoyang Hospital, Capital Medical University, Beijing, China; 2 Laboratory of Immunology for Environment and Health, Shandong Analysis and Test Center, Qilu University of Technology (Shandong Academy of Sciences), Jinan, Shandong China; 3 Department of Immunology and Infectious Disease, The John Curtin School of Medical Research, The Australian National University, Canberra, ACT Australia; 4 Beijing GiantMed Diagnostics Co., LTD, Beijing, China

**Keywords:** Acute retinal necrosis (ARN), T lymphocytes, Cytokine, Varicella zoster virus (VZV), Aqueous humor (AH)

## Abstract

**Background:**

The purpose of this study is to investigate the aqueous humor (AH) T lymphocyte subsets and cytokines of acute retinal necrosis (ARN) to elucidate the immunologic inflammatory features of this disorder.

**Methods:**

Three patients with ARN infected with varicella zoster virus (VZV) who underwent multiple intravitreal injections of ganciclovir were enrolled in this study. The control group consisted of four non-infectious patients with acute anterior uveitis (AAU). Flow cytometric analysis was performed on the lymphocyte subsets from the AH and peripheral blood (PB) samples during the active phase of intraocular inflammation. Five inflammatory cytokines were measured in each AH sample and various clinical characteristics were also assessed.

**Results:**

VZV deoxyribonucleic acid (DNA) was detected by real-time polymerase chain reaction (PCR) in AH from all the ARN patients, who showed higher CD8+ T lymphocytes population in AH than the AAU patients (*p* = 0.006). CD4/CD8 ratios of T lymphocytes and the percentage of CD8 + CD25+ T lymphocytes in AH were significantly lower in ARN than in AAU (*p* = 0.006; *p* = 0.012). In the ARN patients, the percentages of CD4+ and CD8+ T lymphocytes in AH were higher than those found in PB. The percentage of CD4 + CD25+ T lymphocytes in AH was significantly higher than the proportion in PB in the AAU patients (*p* = 0.001). Immunoregulatory cytokine Interleukin-10 in AH was significantly elevated in the ARN patients in comparison with the case of the AAU patients (*p* = 0.036). In ARN, the copy number of VZV DNA in AH positively correlated with the percentage of CD8+ T lymphocytes in AH and negatively correlated with the CD4/CD8 ratio in AH during the course of disease treatment (*p* = 0.009, *r* = 0.92; *p* = 0.039, *r* = − 0.834).

**Conclusion:**

The ARN patients caused by VZV had different intraocular T lymphocyte subsets and cytokines profile than those of the non-infectious patients. High percentages of CD8+ T lymphocytes and low CD4/CD8 T cell ratios may be a potential biomarker for diagnosis of viral-infectious uveitis. T lymphocytes examination at the inflammatory sites has the potential to become a useful research tool for differentiating viral and non-viral uveitis.

## Background

It keeps difficult to find out the cause of uveitis at early stage for clinicians [1]. However, more and more evidence showed that viruses may be the causes for some uveitis with unknown causes previously, such as Posner-Schlossman syndrome [2–4] and Fuchs uveitis syndrome [5, 6]. Since the intraocular cytokine environment and molecular mechanisms and the infiltrating intraocular inflammatory cells or cytokines varied remarkably between viral infectious uveitis and non-viral infectious uveitis [7], it may be a potential way to analyzing the intraocular inflammatory cells and cytokines for clinicians to partially confirm the presumed viral-induced uveitis, even when the real-time polymerase chain reaction (PCR) of specific viruses is negative.

Acute retinal necrosis (ARN) is an infectious viral uveitis syndrome which presents itself as a necrotizing retinitis and may lead to a fatal visual outcome. ARN is most commonly caused by varicella zoster virus (VZV), herpes simplex 1 and 2 (HSV-1, HSV-2), cytomegalovirus (CMV), and infrequently, Epstein-Barr virus (EBV) [8]. VZV is the most common cause, followed by HSV (types 1 and 2) [9].

Apart from the direct cytopathic effect of the pathogenic virus on infected retinal cells, the intraocular T cell response to the triggering virus has been indicated to play a part in the ARN’s local inflammatory response [10, 11]. A previous study reported a high number of CD4+ and CD8+ cells in the vitreous cell population in eyes with ARN mediated by HSV-1 or VZV. CD4+ and cytolytic CD8+ T cell responses make great contributions to restraining virus replication [11], but the immune responses that occur in infectious eyes during the acute infection phase have not been analyzed in detail.

The purpose of this study was to undertake a comprehensive detailed investigation of the types and distribution of T lymphocytes present in aqueous humor (AH) and peripheral blood (PB) in patients with confirmed virus-induced uveitis, i.e., ARN, and provide new insights into the immunological mechanisms that may be important in regulating ocular inflammation following VZV infection. We also compared the differences in the cytokine profiles in the AH from both viral infections and non-infectious patients to aid in the elucidation of mechanisms of the disease. The results may be helpful for clinic to find out a new diagnostic way to differentiate the uveitis between viral causes and autoimmune causes.

## Methods

### Patients

Three consecutive human immunodeficiency virus (HIV)-negative, non-immunosuppressed patients with ARN and four acute anterior uveitis (AAU) patients were recruited between November 2018 and February 2019 from the Ophthalmology Department of Beijing Chaoyang Hospital, which is affiliated to the Capital Medical University. The diagnosis of ARN was confirmed using diagnostic criteria put forward by the American Uveitis Society in 1994 [12]. The etiology of ARN was confirmed based on PCR results for the viral genome of HSV or VZV. The diagnosis of AAU was based on clinical features, including unilateral involvement, acute eye pain, redness, intense photophobia, the presence of keratic precipitates (KPs), aqueous flare, aqueous cells, distorted pupil or posterior synechiae, and elevated intraocular pressure (IOP).

Detailed clinical data, including age, gender, medical history, disease duration from onset to etiological diagnosis, viral species, clinical presentations, and ocular complications were collected from each patient. The purpose of the research and the invasive and therapeutic protocols were explained in detail to all of the patients. All patients underwent anterior chamber paracentesis and AH and PB samples of all the patients were obtained only after informed consent and Ethics Committee approval. This study was conducted according to the principles of the Declaration of Helsinki.

In the ARN group, all the patients received intravenous antiviral treatment and oral antiviral therapy (acyclovir 10 mg/kg thrice daily followed by valacyclovir 1 g 3 times a day or acyclovir 800 mg 5 times a day). Systemic corticosteroids were administered in all patients 48–72 h after the initiation of the antiviral treatment. Samples of AAU patients were taken before the start of the corticosteroids, topical non-steroidal anti-inflammatory drugs (NSAIDs) or topical mydriatic treatment.

### Real-time PCR analysis of AH samples

Genomic DNA of the VZV in the AH was measured using the chickenpox-zoster virus nucleic acid assay kit (Liferiver, Shanghai), and PCR was performed using Multicolor Real-time PCR Detection System (LineGene 9600 Plus, BIOER TECHNOLOGY, Hangzhou). When more than 3000 copies/mL were detected, the value of the viral copy number in the sample was considered significant.

### Immunologic analysis

All of the obtained samples were promptly stored at 4 °C and then brought to the laboratory for cell analysis. AH (100–200 μl) was aspirated and immediately aliquoted into microfuge tube containing cell culture medium to prevent the cells from clumping. The AH samples were centrifuged at 350 g for 5 min. All the samples were then washed and resuspended with staining buffer. Lymphocytes subpopulations were studied by BD FACSCantoII flow cytometry in the AH and PB by using Becton Dickinson monoclonal CD45+, CD3+, CD4+, CD8+, CD15+, CD19+, CD11b+, CD16+, and CD14+ antibodies. According to the high heterogeneity of various lymphocytes, white blood cell regions and each T cell subgroup were circled. Cytokines (IL-8, IL-6, IL-10, vascular endothelial growth factor (VEGF) and IL-1β) were measured by means of CBA with BD FACSCantoII flow cytometry.

### Statistical analysis

Statistical analysis was performed by using SPSS for Windows, version 21.0. Statistical comparisons between the two groups for the CD4/CD8 ratios, CD4+, CD8+ and CD25+ populations were performed by unpaired t-tests or Mann-Whitney U-tests. A *p* value of < 0.05 was considered statistically significant. The Spearman rank correlation coefficient was employed to statistically analyze correlations between cytokines, and between the copy number of VZV DNA and T lymphocytes in the ARN group.

## Results

### Demographic data and clinical characteristics

The demographic data and clinical characteristics of the ARN and the AAU patients were compared (Table 1). All the patients were Chinese. All the ARN patients presented with unilateral necrotizing retinitis, combined with aqueous flare, aqueous cells and vitreous opacity. The occurrence of KPs was recorded in 2 ARN patients. No patient developed retinal detachment. All the ARN patients had VZV infection confirmed by PCR. All the 4 AAU patients had coexisting autoimmune disorders or serum autoantibodies. Two patients had coexisting rheumatic arthritis (RA), 1 patient had coexisting ankylosing spondylitis (AS), and 2 patients had coexisting serum human leukocyte antigen (HLA)-B27 positive. The AAU patients also presented with aqueous flare, aqueous cells and KP, but none presented with vitreous opacity or retinal lesion. Scleritis accompanied with ocular pain was found in 1 AAU patient. AH and PB samples of all the AAU patients were obtained only once at the acute phase of the disease. In the ARN group, the AH and PB of patients 1 and 3 were collected at the acute phase, 1 week and 2 weeks after treatment, respectively, and a total of 3 samples of every patient were collected. The data for Patient 2 in the ARN group were collected once at the acute phase.

**Table 1:** Demographic and clinical characteristics of the patients with ARN and AAU

		Age	BCVA	IOP (mmHg)	Keratic Precipitate	Aqueous Flare	Aqueous Cell	Vitreous Opacity	Ocular Pain	Necrotizing Retinits	Retinal Detachment	VZV DNA	Autoimmune Disease & Auto-Ab
ARN	Patient 1	45	0.09	25	++	+++	+++	+++	–	+	–	1.06E+ 07	–
	Patient 2	18	0.6	20	+	++	+++	+++	–	+	–	1.21E+ 05	–
	Patient 3	41	0.12	15	–	++	+++	+++	–	+	–	1.10E+ 06	–
AAU	Patient 4	54	0.2	7	–	+	+++	–	–	–	–	–	RA
	Patient 5	32	FC	27	+	++++	+++	–	+	–	–	–	HLA-B27+
	Patient 6	38	0.2	17	+	+	+++	–	–	–	–	–	AS, HLA-B27+
	Patient 7	30	0.4	11	++	++	+++	–	–	–	–	–	RA

*ARN* Acute Retinal Necrosis, *AAU* Acute Anterior Uveitis, *BCVA* Best Corrected Visual Acuity, *IOP* Intraocular Pressure, *VZV* Varicella Zoster Virus, *DNA* Deoxyribonucleic Acid, *RA* Rheumatoid Arthritis, *HLA* Human Leukocyte Antigen, *AS* Ankylosing Spondylitis, *Auto-Ab* Auto-Antibodies

### Comparison of flow cytometric analyses of aqueous humor and peripheral blood samples

**Fig. 1:**
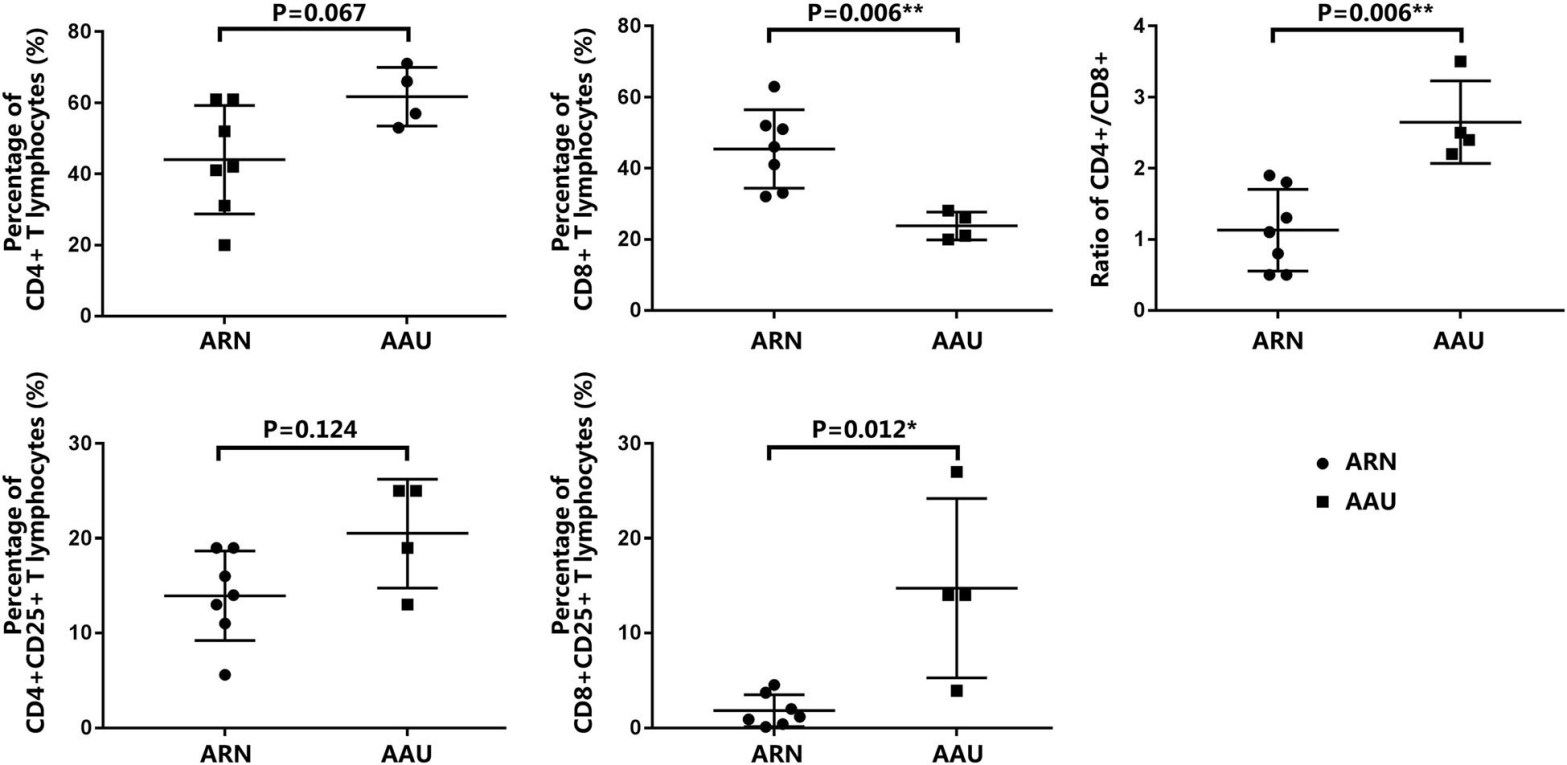
Comparison of percentage of CD4+, CD8+, CD4 + CD25+ and CD8 + CD25+ T lymphocytes and ratio of CD4/CD8 in AH between ARN and AAU patients. The comparisons between the ARN and AAU groups for the CD4/CD8 ratios, CD4+, CD8+ and CD25+ populations were performed by Mann-Whitney U-tests

Figure 1 presents the comparison results of the flow cytometric analyses of aqueous humor and peripheral blood samples according to the etiology of uveitis. The percentages of lymphocytes in the AH and PB of the ARN patients were compared with those in the AH and PB of the AAU patients. The percentage of AH CD8+ T lymphocytes was higher in the ARN patients than in the AAU patients (*p* = 0.006). The ARN group showed a lower percentage of intraocular CD4+ T cells than the AAU group, although no significant difference was found (*p* = 0.067). The percentages of CD4+ and CD8+ T lymphocytes in the PB samples were no different between the ARN and AAU groups. The CD4/CD8 ratios of lymphocytes obtained from the AH samples of the ARN patients were significantly lower (*p* = 0.006) than those in the AH of the AAU group. All the ARN patients had a CD4/CD8 ratio of less than 2.0. However, no significant difference was observed in the CD4/CD8 ratio of peripheral lymphocytes between the ARN and AAU groups (*p* = 0.291). In addition, the percentage of CD8 + CD25+ T lymphocytes obtained from the AH in ARN patients was significantly lower (*p* = 0.012) than that of AAU patients. In the percentage of CD4 + CD25+ T lymphocytes, no significant difference was found (*p* = 0.124) between the AH samples from the ARN and AAU patients. As to the PB samples, no significant difference was found in the percentages of CD4 + CD25+ and CD8 + CD25+ T lymphocytes between the ARN and AAU groups.

**Fig. 2:**
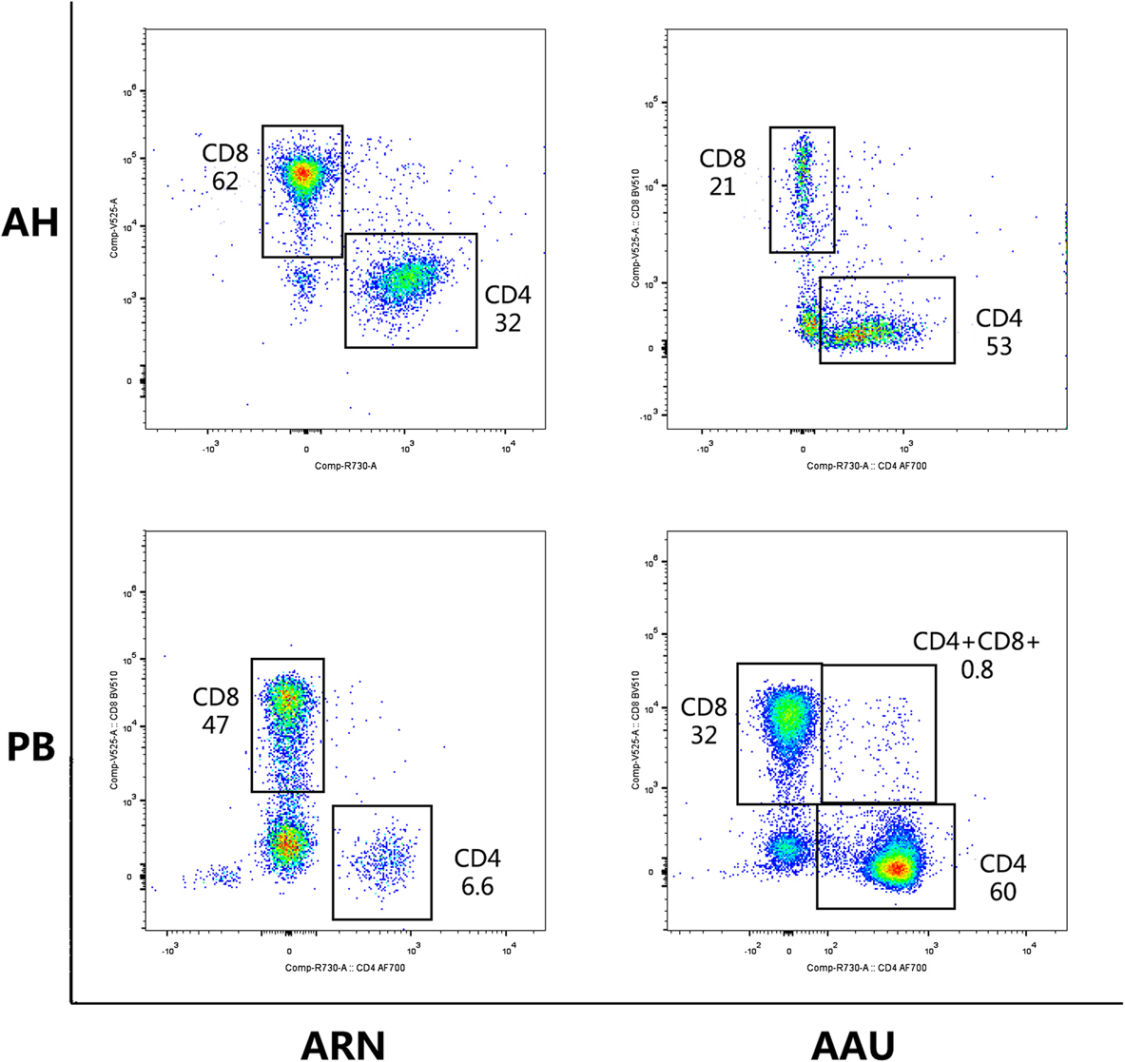
Representative dot plot diagrams showing the percentage of CD4+ and CD8+ T cell subsets among the PB and AH cells in patients with acute retinal necrosis (ARN) and acute anterior uveitis (AAU). The percentage of CD8+ T cell in the AH was elevated in ARN patients

Table 2 shows the comparison of the flow cytometric analyses of the AH and PB samples obtained from the ARN and AAU groups. In the ARN group, the percentages of CD4+, CD4 + CD25+ and CD8+ T lymphocytes in AH were higher than those found in PB, although no statistically significant differences were found between the AH samples and PB samples in the percentages of CD4+, CD8+, CD4 + CD25+ and CD8 + CD25+ T lymphocytes. For the AAU patients, the percentages of CD4 + CD25+ and CD8 + CD25+ T lymphocytes in AH were both higher than those in PB, but no statistical significance was found in the percentage of CD8 + CD25+ T lymphocytes. The AH samples of the AAU patients had a higher percentage of CD4 + CD25+ T lymphocytes than that of the PB samples of the AAU patients (*p* = 0.001). No marked difference in the percentages of CD4+ and CD8+ T lymphocytes was observed between the two types of samples. No significant difference in CD4/CD8 lymphocyte ratios between AH and PB was found in both the ARN and AAU patients. Representative flow cytometric data of the AH and PB samples from the ARN and AAU patients are shown in Fig. 2.

**Table 2:** Comparison of percentages of T lymphocyte subsets of the ARN and AAU patients

	ARN			AAU		
	AH	PB	*p* Value^†^	AH	PB	*p* Value^†^
CD4+ (%), median (P25, P75)	50.0 (41.0, 61.0)	46.0 (40.0, 50.0)	0.364	61.5 (54.0, 69.8)	56.5 (32.0, 66.0)	0.354
CD4 + CD25+ (%), median (P25, P75)	14.0 (11.0, 19.0)	7.9 (4.1, 13.0)	0.074	22.0 (14.5, 25.0)	3.3 (1.95, 5.3)	0.001*
CD8+ (%), median (P25, P75)	46.0 (33.0 52.0)	43.0 (41.0, 47.0)	0.662	23.5 (20.3, 27.5)	36.5 (26.8, 55.3)	0.091
CD8 + CD25+ (%), median (P25, P75)	1.2 (0.4, 3.7)	1.5 (1.4, 2.3)	0.753	14.0 (6.4, 23.8)	1.9 (0.85, 10.9)	0.125
CD4+/CD8+, median (P25, P75)	1.1 (0.8, 1.8)	1.1 (0.9, 1.2)	0.703	2.45 (2.25, 3.25)	1.6 (0.63, 2.5)	0.106

*ARN* Acute Retinal Necrosis, *AAU* Acute Anterior Uveitis, *AH* Aqueous Humor, *PB* Peripheral Blood

^*^P < 0.05

^†^Mann–Whitney U-test

### Differences of cytokine expression between the ARN and AAU patients

The expression of cytokines in AH of the ARN and AAU patients was summarized in Table 3. In the AH of the ARN patients, cytokines IL-6 and IL-10 were elevated compared with those of the AAU patients, although no significant difference was found in the increase of IL-6 (IL-10: *p* = 0.036, IL-6: *p* = 0.727). There were no significant differences between the two groups as to AH concentration of IL-8, VEGF and IL-1β.

**Table 3:** Cytokines levels in the AH of ARN and AAU patients

	ARN	AAU	*P* Value^†^
IL-8 (pg/ml), median (P25, P75)	1118.4 (467.9, 2057.3)	2287.3 (294.6, 4457.8)	0.373
IL-6 (pg/ml), median (P25, P75)	7673.3 (2071.5, 26,725.2)	3963.3 (3037.8, 8815.8)	0.727
IL-10 (pg/ml), median (P25, P75)	66.5 (43.9, 178.8)	11.7 (7.9, 21.3)	0.036*
VEGF (pg/ml), median (P25, P75)	234.8 (93.0, 604.4)	719.2 (442.4, 994.9)	0.286
IL-1β (pg/ml), median (P25, P75)	4.7 (0.0, 6.9)	11.0 (0.0, 39.6)	0.397

*ARN* Acute Retinal Necrosis, *AAU* Acute Anterior Uveitis, *AH* Aqueous Humor, *PB* Peripheral Blood, *IL-8* Interleukin-8, *IL-6* Interleukin-6, *IL-10* Interleukin-10, *VEGF* Vascular Endothelial Cell Growth Factor, *IL-1β* Interleukin-1β

^*^P < 0.05

^†^Mann–Whitney U-test

### Correlation between the copy number of VZV DNA and the T lymphocytes in AH

**Fig. 3:**
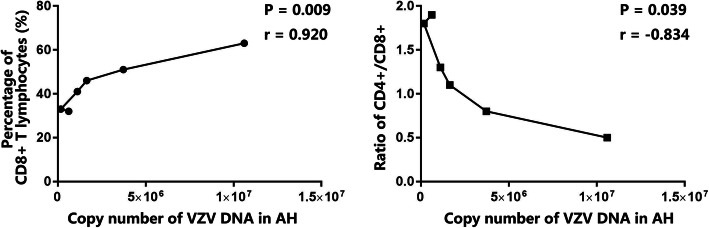
The correlation between T lymphocytes and VZV DNZ load in AH. A graph showing the copy number of VZV DNA in AH positively correlated well with the percentage of the CD8+ T lymphocytes in AH (*p* = 0.009, *r* = 0.92). B graph showing the CD4/CD8 ratio of AH negatively correlated with the copy number of VZV DNA in the ARN patients (*p* = 0.039, *r* = − 0.834). The Sperman’s correlation and the *p* value are indicated at the figure

We then analyzed the potential correlations between the decreased copy number of VZV DNA after antiviral treatment and the percentages of CD4+, CD8+, CD4 + CD25+, and CD8 + CD25+ T lymphocytes and CD4/CD8 ratio. Table 4 shows the significant decrease of aqueous viral load in 2 ARN patients. One week after antiviral treatment, the aqueous viral load of Patient 1 decreased from 1.06*10^7^ copies/mL to 3.72*10^6^ copies/mL, and decreased to 1.64*10^6^ copies/mL after 2 weeks of treatment. As to Patient 3, the aqueous viral load decreased from 1.10*10^6^ copies/mL to 1.65*10^5^ copies/mL after 1 week of treatment, and slightly increased to 6.14*10^5^ copies/mL after 2 weeks of treatment. Further correlation analyses showed that the copy number of VZV DNA in AH positively correlated well with the percentage of the CD8+ T lymphocytes in AH (*p* = 0.009, *r* = 0.92; Fig. 3). Although the percentage of CD4+ T lymphocytes in AH increased after antiviral treatment, the tendency of CD4+ T cells was not significantly correlated to the aqueous VZV load (*p* = 0.286). Furthermore, it was found that the CD4/CD8 ratio of AH negatively correlated with the copy number of VZV DNA in the ARN patients (*p* = 0.039, *r* = − 0.834; Fig. 3). No significant correlation between the aqueous VZV load and the percentages of CD4 + CD25+ and CD8 + CD25+ T lymphocytes was observed (CD4 + CD25+: *p* = 0.414; CD8 + CD25+: *p* = 0.551). These findings collectively imply that the percentage of CD8+ T lymphocytes and the ratio of CD4/CD8 in AH may be a candidate biomarker for active intraocular viral infectious diseases, especially in ARN.

**Table 4:** Changes of the T lymphocyte subsets and the copy number of VZV DNA in AH after treatment

		CD4+ (%)	CD4 + CD25+ (%)	CD8+ (%)	CD8 + CD25+ (%)	CD4/CD8	VZV DNA (copies/mL)
Patient 1	Acute Phase	31	19	63	0.9	0.5	1.06E+ 07
	1w after treatment	41	14	51	0.4	0.8	3.72E+ 06
	2w after treatment	20	11	46	0.1	1.1	1.64E+ 06
Patient 3	Acute Phase	52	13	41	3.7	1.3	1.10E+ 06
	1w after treatment	61	16	33	1.2	1.8	1.65E+ 05
	2w after treatment	61	19	32	2.0	1.9	6.14E+ 05

*ARN* Acute Retinal Necrosis, *AAU* Acute Anterior Uveitis, *VZV* Varicella Zoster Virus, *DNA* Deoxyribonucleic Acid, *DNA* Deoxyribonucleic Acid

## Discussion

This study described the immunological features of the AH and PB T lymphocytes population in patients with viral infections or non-infectious uveitis. It also analyzed the mainly inflammatory cytokines of the AH samples of those patients.

The present study reported that there are intrinsic differences in both the T lymphocytes subsets and cytokines within the AH when comparing the VZV mediated ARN patients and the AAU patients. VZV is a double-stranded DNA virus belonging to the herpesviridae family. Among the human herpes viruses, HSV-1 and 2 and VZV are neurotropic, i.e., they establish latent infection in the peripheral nervous system and the viral genome is retained in peripheral sensory ganglia throughout the host’s lifespan [13, 14]. Most ARN patients are immunocompetent, and typical cases of ARN are uncommon in immunocompromised individuals [14]. VZV infection provides stimuli to the antigen-specific cell-mediated immunity essential to recovering from primary infection, to containing VZV reactivation, and to protecting against virus. It was reported that T cells predominate among the inflammatory cells located in AH of ARN patients [15]. The present study described the immunologic profile of AH T lymphocytes in ARN patients characterized by a high percentage of CD8+ T lymphocytes and low CD4/CD8 ratio. It was found that CD8+ T lymphocytes infiltration into the AH was higher in patients with ARN than patients with AAU, and this is consistent with previous reports [16, 17]. In comparison with the non-infectious patients, significant increase of CD8+ cell population and decrease of CD4/CD8 ratio in AH during the acute phase of viral infection were observed in the viral infectious patients. Previous studies found that large immune infiltrates consisted of CD8+ T cells following VZV activation in human ganglia [14, 18]. These results were similar to those found in previous research, providing further support to the hypothesis that changes in T lymphocytes subpopulations, including a higher percentage of CD8+ T cells and a low CD4/CD8 ratio, are associated with viral activation. After antiviral treatment, the symptoms of the ARN patients relieved, manifesting in the form of reduced ocular inflammation and improved visual acuity. The copy number of VZV DNA in AH in the ARN patients significantly decreased after treatment. Correlation analyses showed that the copy number of VZV DNA in AH positively correlated with the percentage of CD8+ T lymphocytes in AH and negatively correlated with the CD4/CD8 ratio in AH. Importantly, this study also showed that after treatment the percentage of CD8+ lymphocytes in the ARN patients was even higher than that in the AAU patients. Similarly, even after antiviral treatment, the ratio of CD4/CD8 was lower in the ARN group than that in the AAU group. Therefore, a high percentage of CD8+ T lymphocytes and a low CD4/CD8 T cell ratio may be a potential biomarker, serving as clinically useful indicators to evaluate the severity of intraocular immune inflammatory response by which the prognosis of ARN can be forecasted. The identification and analysis of T lymphocytes from sites of intraocular inflammation can help investigate the roles of infiltrating T lymphocytes in the pathogenesis of uveitis with different etiologies.

In the uveitis associated with systemic and intraocular infections, it is unclear whether the T lymphocytes enter the eye as part of a peripheral immune response, or there is a passive extravasation of T lymphocytes enter the eye during a breakdown of the blood-ocular barrier. In line with previously published data, the percentages of CD4+ and CD8+ T lymphocytes in AH were higher than those found in PB [11]. In this study, compared with the non-infectious patients, ARN manifests peculiar focal lymphocytosis characterized by an elevated percentage of CD8+ T lymphocytes and a corresponding decreased CD4/CD8 ratio in AH. However, the changes of CD8+ T lymphocytes and CD4/CD8 patterns were observed only in AH but not in PB. The results suggest that there may be a particular mechanism attracting T lymphocytes into the eye, or the blood-aqueous barrier may channel the selective infiltration of activated T cells into uveitis patients’ ocular tissues and AH. Another possible explanation for this is that the ARN patients with ocular inflammation only, but without other systemic symptoms, are too constrained to enable detection of whatever alterations in the blood’s cell phenotypes. PB is not the perfect sample for research on immune abnormalities in intraocular inflammation in that it cannot represent the immune inflammatory processes taking place within the eye. Further detailed studies are necessary to investigate the immune function of intraocular T lymphocyte subsets and immunologic abnormalities in viral infectious uveitis, or to study whether there is a characteristic T lymphocyte population in the AH of viral infection patients.

CD4 + CD25+ T cells have been shown to be one distinct subset of regulatory T cells for the prevention of autoimmunity [19]. Several studies have shown that regulatory CD4 + CD25+ T cells contribute much to controlling immune homeostasis, to maintaining self-tolerance, and to preventing autoimmunity, as impaired CD4 + CD25+ T cell activity can cause autoimmune disease [20, 21]. There was predominant CD4 + CD25+ T cell enrichment in the inflammatory sites during the disease course and their accumulation correlated with disease resolution [22]. In this study, the CD25+ T lymphocytes comprised 20.5% of the CD4+ T lymphocytes in AH in the AAU patients, who had significantly increased CD4 + CD25+ T lymphocytes in AH compared with PB. All the AAU patients had coexisting autoimmune disorder or serum autoantibodies, and the anterior uveitis of those patients was occurring in association with systemic diseases. In the AAU patients, the enrichment of regulatory CD4 + CD25+ T lymphocytes in AH may suppress the activation and proliferation of T cells to regulate local inflammatory responses [23]. However, there were collaborative roles for several mechanisms requiring both cell-cell contact and cytokines production in the identification of the quantity and function of CD4 + CD25+ T cells in the inflammatory sites and peripheral immune sites [24]. Further mechanism research is needed to investigate the regulatory mechanism and immunosuppressive activity of regulatory CD4 + CD25+ T lymphocytes.

Chemokines are unnegligible inflammatory mediators excreted by inflammatory and immune cells during ocular inflammation [25]. The intraocular concentration of IL-10 is very useful for etiology diagnosis of uveitis, especially in intraocular lymphoma (IOL) [26]. Previous studies have also reported that IL-10 was highly elevated in herpetic and noninfectious uveitis [7, 27]. IL-10 is an immunosuppressive regulatory cytokine and can be produced by T lymphocytes. It has been suggested that IL-10 downregulates the immune responses in order to moderate T cell mediated immune reaction and regulate the extent of tissue damage [28, 29]. The high expression of intraocular IL-10 in the ARN patients was in keeping with previous research [7, 26]. However, the high IL-10 level in AH of the ARN patients seems paradoxical regarding the severe course of the intraocular inflammation. One possible reason is that the expression of IL-10 is not sufficient to suppress the harmful inflammatory immune response. Alternatively, in addition to the immune responses downregulated by IL-10, other potential inflammatory response mechanisms may contribute in combination to the damage. Because the cytokine and chemokine expression patterns are constituents of a very complex regulatory network that depends on many environmental stimuli, they are likely to vary remarkably over the progression of the disease. Therefore, the effect of cytokines on the T lymphocytes necessitates thorough investigation if cytokine immunology therapy is to be employed in future. Combined with changes of intraocular cytokines, immune cells profiles in AH seem to provide insights into the severity and stage of the inflammatory process. Our results show that through cytometric analysis of AH, the immunologic information of this disease was useful for the differential diagnosis of intraocular viral infection. It helps improve our ability to classify viral infection from other uveitis, which could enable an early and less-invasive diagnosis.

Undeniably, this study has its limitations. First, the number of the ARN and AAU patients enrolled in the study was not sufficient, and therefore future research needs to further enlarge the sample size to analyze the changes of T lymphocyte subsets in these diseases. Further studies will provide detailed information on immunopathologic mechanisms of lymphocytes in AH from different types of uveitis. Second, this study focused on the distribution of T lymphocytes subsets in AH and PB of ARN and AAU patients, but did not analyze the function of those cells. Further thorough studies are necessitated to investigate the detailed T lymphocyte subsets and functional properties of intraocular infiltrating T lymphocytes in uveitis patients in order to elucidate underlying immunopathogenic mechanisms of this disorder. Third, the follow-up period for the patients with antiviral treatment was not long enough, but future longitudinal follow-up studies in ARN patients are expected to overcome this shortcoming, and hopefully, they may provide precious insights into the progression of immune inflammation in ARN.

## Conclusions

In conclusion, the patients with ARN caused by VZV in this study had a higher percentage of CD8+ T lymphocytes and a lower CD4/CD8 ratio in AH compared with the control subjects suffering non-infectious uveitis. The percentage of the CD8+ T lymphocyte subset and the ratio of the CD4/CD8 in AH from the ARN patients significantly correlated to the aqueous VZV load during the course of disease treatment. AH cell profiles might be discriminative for the diagnosis of viral-infectious and immune-mediated uveitis, and AH cell examination has the potential to become a useful research tool for investigating various uveitis entities.

## Data Availability

The datasets used during the current study are available from the corresponding author on reasonable request.

## Abbreviations

AH: Aqueous humor
ARN: Acute retinal necrosis
VZV: Varicella zoster virus
AAU: Acute anterior uveitis
PB: Peripheral blood
PCR: Polymerase chain reaction
IL: Interleukin
HSV: Herpes simplex virus
CMV: Cytomegalovirus
EBV: Epstein-Barr virus
KPs: Keratic precipitates
IOP: Intraocular pressure
